# Parsing heterogeneity within dementia with Lewy bodies using clustering of biological, clinical, and demographic data

**DOI:** 10.1186/s13195-021-00946-w

**Published:** 2022-01-21

**Authors:** Carla Abdelnour, Daniel Ferreira, Marleen van de Beek, Nira Cedres, Ketil Oppedal, Lena Cavallin, Frédéric Blanc, Olivier Bousiges, Lars-Olof Wahlund, Andrea Pilotto, Alessandro Padovani, Mercè Boada, Javier Pagonabarraga, Jaime Kulisevsky, Dag Aarsland, Afina W. Lemstra, Eric Westman

**Affiliations:** 1 Research Center and Memory Clinic, Ace Alzheimer Center Barcelona, Institut Català de Neurociències Aplicades, Universitat Internacional de Catalunya-Barcelona, Centro de Investigación en Red-Enfermedades Neurodegenerativas (CIBERNED), Barcelona, Spain; 2 Department of Medicine of the Universitat Autònoma de Barcelona, Barcelona, Spain; 3 Division of Clinical Geriatrics, Centre for Alzheimer Research, Department of Neurobiology, Care Sciences, and Society, Karolinska Institutet, Stockholm, Sweden; 4 Alzheimer Center Amsterdam, Department of Neurology, Amsterdam Neuroscience, Vrije Universiteit Amsterdam, Amsterdam UMC, Amsterdam, The Netherlands; 5 Department of Psychology, Sensory Cognitive Interaction Laboratory (SCI-lab), Stockholm University, Stockholm, Sweden; 6 Centre for Age-Related Medicine, Stavanger University Hospital, Stavanger, Norway; 7 Department of Radiology, Stavanger University Hospital, Stavanger, Norway; 8 Department of Electrical Engineering and Computer Science, University of Stavanger, Stavanger, Norway; 9 Department of Neuroscience, Karolinska Institutet, Stockholm, Sweden; 10 Department of Radiology Karolinska University Hospital, Stockholm, Sweden; 11 Service, Memory Resources and Research Centre, University Hospital of Strasbourg, Strasbourg, France; 12 Team IMIS/Neurocrypto, French National Center for Scientific Research, ICube Laboratory and Fédération de Médecine Translationnelle de Strasbourg (FMTS), University of Strasbourg, Strasbourg, France; 13 Centre Mémoire, de Ressources et de Recherche d’Alsace (Strasbourg-Colmar), Strasbourg, France; 14 Laboratory of Biochemistry and Molecular Biology, CNRS, Laboratoire de Neurosciences Cognitives et Adaptatives, UMR7364, University Hospital of Strasbourg, Strasbourg, France; 15 Neurology Unit, Department of Clinical and Experimental Sciences, University of Brescia, Brescia, Italy; 16 Movement Disorders Unit, Neurology Department, Hospital de la Santa Creu i Sant Pau. Biomedical Research Institute (IIB-Sant Pau), Centro de Investigación en Red-Enfermedades Neurodegenerativas (CIBERNED), Barcelona, Spain; 17 Institute of Psychiatry, Psychology and Neuroscience, King’s College London, London, UK; 18 Department of Neuroimaging, Centre for Neuroimaging Sciences, Institute of Psychiatry, Psychology and Neuroscience, King’s College London, London, UK

**Keywords:** Dementia with Lewy bodies, Alzheimer’s disease, Factorial analysis, Hierarchical clustering, Biomarkers, Heterogeneity

## Abstract

**Background:**

Dementia with Lewy bodies (DLB) includes various core clinical features that result in different phenotypes. In addition, Alzheimer’s disease (AD) and cerebrovascular pathologies are common in DLB. All this increases the heterogeneity within DLB and hampers clinical diagnosis. We addressed this heterogeneity by investigating subgroups of patients with similar biological, clinical, and demographic features.

**Methods:**

We studied 107 extensively phenotyped DLB patients from the European DLB consortium. Factorial analysis of mixed data (FAMD) was used to identify dimensions in the data, based on sex, age, years of education, disease duration, Mini-Mental State Examination (MMSE), cerebrospinal fluid (CSF) levels of AD biomarkers, core features of DLB, and regional brain atrophy. Subsequently, hierarchical clustering analysis was used to subgroup individuals based on the FAMD dimensions.

**Results:**

We identified 3 dimensions using FAMD that explained 38% of the variance. Subsequent hierarchical clustering identified 4 clusters. Cluster 1 was characterized by amyloid-β and cerebrovascular pathologies, medial temporal atrophy, and cognitive fluctuations. Cluster 2 had posterior atrophy and showed the lowest frequency of visual hallucinations and cognitive fluctuations and the worst cognitive performance. Cluster 3 had the highest frequency of tau pathology, showed posterior atrophy, and had a low frequency of parkinsonism. Cluster 4 had virtually normal AD biomarkers, the least regional brain atrophy and cerebrovascular pathology, and the highest MMSE scores.

**Conclusions:**

This study demonstrates that there are subgroups of DLB patients with different biological, clinical, and demographic characteristics. These findings may have implications in the diagnosis and prognosis of DLB, as well as in the treatment response in clinical trials.

**Supplementary Information:**

The online version contains supplementary material available at 10.1186/s13195-021-00946-w.

## **Background**

The current diagnosis of probable dementia with Lewy bodies (DLB) is based on the presence of cognitive impairment, sufficient to impact patients’ ability to perform activities of daily living. In addition, at least two of the following core clinical features must be present: parkinsonism, recurrent visual hallucinations, cognitive fluctuations, and/or rapid eye movement (REM) sleep behavior disorder (RBD) [1]. These core clinical features often manifest in different combinations at the time of diagnosis or during the course of the disease, increasing the clinical heterogeneity within probable DLB. Previous studies have addressed part of this heterogeneity by investigating subgroups of patients with certain core clinical features or different rates of clinical progression [2, 3]. However, extending these analyses to biological features of the disease is warranted to elucidate the pathophysiology underlying the heterogeneity within probable DLB.

Although very few studies have directly addressed the biological heterogeneity in DLB, there is an increasing interest in how Alzheimer’s disease (AD)-related pathology contributes to clinical presentation in DLB. Part of the heterogeneity in DLB could be related to concomitant AD pathology, which is present in more than 50% of DLB patients in neuropathological studies [4, 5] and in around 30% in in vivo biomarker studies [6, 7]. A recent multi-center study in DLB patients showed that amyloid-β pathology influences cognitive performance, whereas tau affects clinical presentation through an association with lower frequency of parkinsonism and probable RBD [8]. In other cohorts, DLB patients with positive AD biomarkers more frequently showed visual hallucinations [9]. Biological heterogeneity can also be studied through structural magnetic resonance imaging (MRI). A recent study investigated four atrophy subtypes in DLB, and concluded that the pattern with prominent cortical atrophy and sparing of the hippocampus was the most common subtype in probable DLB [10]. However, how all these dimensions of heterogeneity inter-relate with each other is completely unknown. Perhaps subgroups with distinct CSF profiles, atrophy patterns and clinical phenotypes are present. Multimodal subtyping studies are urgently needed to address this question, but such studies are lacking so far [11].

The goal of the current study was to parse the heterogeneity within probable DLB by using a multimodal subtyping method applied on the combination of CSF biomarkers, structural MRI, and clinical and demographic measures. We gathered data from a large multi-center cohort of patients with probable DLB (*N* = 107). Firstly, we identified subgroups of patients with factorial analysis and multimodal clustering. Secondly, we characterized the resulting subgroups across key CSF, MRI, clinical, and demographic measures.

## Methods

### Participants

Participants were selected from the European DLB consortium (E-DLB) [12]. The E-DLB consortium archives data from 40 centers across Europe, including patients with probable DLB, Parkinson’s disease with dementia, or AD. For the current study, we included patients with probable DLB from the E-DLB centers that had MRI and CSF biomarkers available. Six centers satisfied these criteria, including the Alzheimer Center Amsterdam, Amsterdam UMC (Amsterdam, the Netherlands, *n* = 38); Day Hospital of Geriatrics, Memory Resource and Research Centre (Strasbourg, France, *n* = 38); Karolinska Institutet (Stockholm, Sweden, *n* = 17); University of Brescia (Brescia, Italy, *n* = 6); Ace Alzheimer Center Barcelona (Barcelona, Spain, *n* = 5); and Stavanger University Hospital (Stavanger, Norway, *n* = 3). A total of 107 probable DLB patients were included.

The diagnostic procedure and clinical examinations are described elsewhere [13]. Briefly, the diagnosis was made according to the 2005 International Consensus Criteria for probable DLB [14], based on detailed history and clinical assessment including physical, neurological, and psychiatric examinations performed by a licensed neurologist. The criteria from 2005 were used because many of the patients were assessed prior to the 2017 International Consensus Criteria [15]. Exclusion criteria were patients with acute delirium, terminal illness, stroke, psychotic or bipolar disorder, craniocerebral trauma, or a major neurological illness other than dementia. All centers recorded whether patients fulfilled the criteria for parkinsonism, visual hallucinations, cognitive fluctuations, and a clinical history of probable RBD. Data about clinical core features were classified into present or absent in order to standardize the information across centers, for statistical analyses. The Mini-Mental State Examination (MMSE) was scored as a measure of global cognition [16].

### Magnetic resonance imaging

MRI scanners and protocols used at each center are described in Supplementary Table 1 (Additional file 1). Due to variability in MRI scanners and protocols, we favored visual rating scales by an experienced neuroradiologist (L.C.), rather than the application of automated methods for regional brain atrophy. The neuroradiologist was blind to any clinical information including diagnosis. Regional atrophy was assessed with three visual rating scales based on T1-weighted images as detailed elsewhere [17]. Briefly, atrophy in the medial temporal lobe was assessed with the MTA scale [18]; atrophy in the posterior cortex was assessed with the PA scale [19]; and atrophy in the frontal lobe was assessed with the GCA-F scale [20]. In the three visual rating scales, a score of zero denotes no atrophy, whereas scores from one to three/four indicate an increasing degree of atrophy. MTA analysis was based on coronal reconstructions, GCA-F on axial reconstructions, and PA on reconstructions from all three planes. Our neuroradiologist (L.C.) has previously demonstrated excellent intra-rater reliability in 120 random cases: weighted kappa values of 0.94 and 0.89 for MTA in left and right hemispheres, respectively; 0.88 for posterior atrophy (PA); and 0.83 for global cortical atrophy scale–frontal subscale (GCA-F) [17]. The same neuroradiologist assessed white matter hyperintensities (WMHs) on axial FLAIR images, as a marker of cerebrovascular disease, using the Fazekas scale [21]. Briefly, the Fazekas scale grades WMHs as 0 (i.e., absence of WMHs), 1 (i.e., punctate WMHs), 2 (i.e., early confluent WMHs), and 3 (i.e., WMHs in large confluent areas). Fazekas scores were classified into low (Fazekas scores 0 or 1) and high (Fazekas scores 2 or 3) WMH burden, as in previous studies [22, 23].

### Cerebrospinal fluid biomarkers

Amyloid-β and tau neurofibrillary tangles were assessed through CSF levels of Aβ42 and phosphorylated tau (p-tau) at threonine 181. We also included total tau CSF levels as a marker of unspecific neurodegeneration. All CSF analyses were performed locally following standard routines. Methods for CSF sampling, analysis, and cut-off values for each center are described elsewhere [6, 24] and detailed in Supplementary Table 2 (Additional file 1). Briefly, INNOTEST enzyme-linked immunosorbent assays (ELISAs) from Fujirebio, Ghent, Belgium, were used for total tau and p-tau biomarkers in all samples and for Aβ42 in 101 samples. ELISA kits from Biosource Europe S.A were used to analyze Aβ42 in the remaining 6 samples. To further standardize the information on CSF biomarkers across centers, CSF Aβ42, p-tau, and total tau values were classified as normal (-) or abnormal (+) using well-established center-specific cut-off points, as described in previous E-DLB studies [6, 24]. The frequency of abnormal CSF biomarker values was compared across DLB subgroups. In addition, subgroup characterization was also done on the basis of a CSF AD profile, following the current AT(N) classification framework to define AD biologically [25]. Briefly, abnormal levels of the Aβ42 biomarker alone were considered as indicative of an AD pathological change (A+T-). Abnormal levels of the p-tau biomarker were considered as indicative of AD pathology when in combination with abnormal levels of the Aβ42 biomarker (A+T+) and considered as a non-AD pathologic change when in combination with normal levels of the Aβ42 biomarker (A-T+). In the context of current discussions about the role and meaning of tau pathology in DLB [26–28], and in consistence with our previous study [8], we described this non-AD pathologic change as amyloid-independent tau-pathology in DLB patients.

### Statistical analysis

**Fig. 1:**
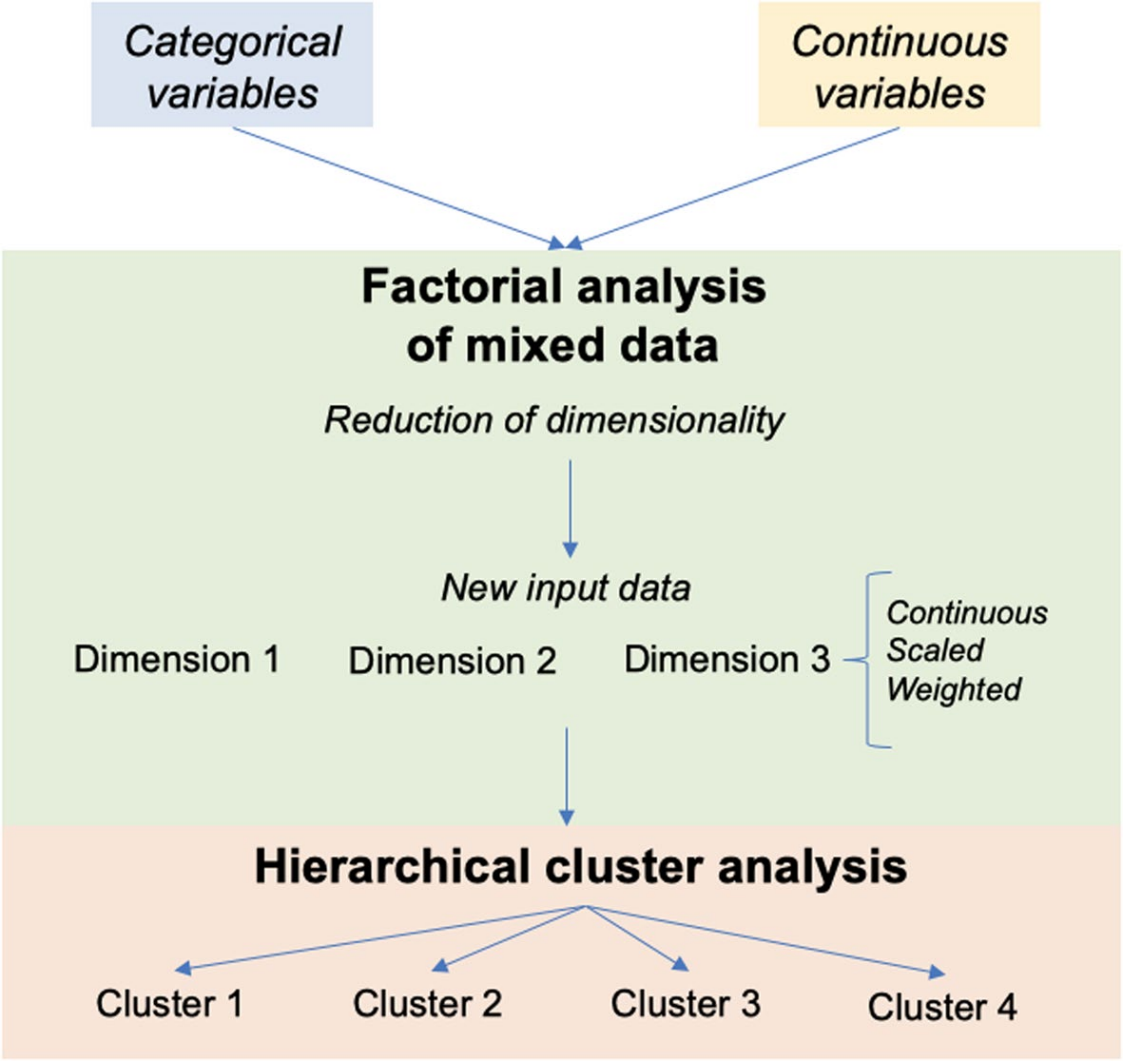
Methodological design. Overview of factorial analysis of mixed data (FAMD) and hierarchical clustering analysis

The main aim of this study was to parse DLB heterogeneity and identify different subgroups of patients based on CSF biomarkers, regional brain atrophy, and key demographic and clinical measures. This was done in two steps as explained below and depicted in Fig. 1.

In the first step, we aimed to identify the latent dimensions/components in the data that determine DLB heterogeneity. Since our data included both continuous and categorical variables, we used a multivariate method for data analysis called factorial analysis of mixed data (FAMD) [29]. The main strength of FAMD is that it accommodates both quantitative and qualitative data simultaneously. FAMD works as a principal component analysis for quantitative data and as a multiple correspondence analysis for qualitative data [29]. In our FAMD model, age, years of education, MMSE scores, and disease duration were included as continuous variables, and sex (male vs. female), CSF Aβ42, p-tau and total tau levels, MTA, PA, and GCA-F scales (normal vs. abnormal), and parkinsonism, visual hallucinations, cognitive fluctuations, and probable RBD (absent vs. present) were included as categorical variables. Fazekas scores (low vs. high WMH burden) were not included in the FAMD model and subsequent cluster analysis due to missing data, but they were used to characterize the resulting subgroups, post hoc.

In the second step, we aimed to classify patients into subgroups using a cluster analysis based on the dimensions provided by the FAMD model. Cluster analysis was not applied directly on the original data because variables come in different scales and have a mixed nature (quantitative and qualitative). Instead, the output of the FAMD model is a suitable input for cluster analysis because it is scaled (all dimensions have the same scale) and continuous, and the high dimensionality of the original data is reduced to a few latent dimensions (three in our study, please see in the “Results” section). Furthermore, the original variables are represented with different weights in the dimensions, according to their contribution to the dimensions and the portion of variance explained by each dimension. We applied an agglomerative hierarchical clustering algorithm with Ward’s linkage method [30]. This clustering method starts by assigning every DLB patient to one cluster and sequentially combines pairs of clusters at each step while minimizing the sum of square errors from the cluster mean. The algorithm continues merging DLB patients into clusters until all the patients form a single group. We identified the optimal number of clusters by using the Calinski-Harabasz criterion [31] and by visual inspection of the dendrogram from the agglomerative hierarchical clustering.

We characterized the resulting subgroups using one-way ANOVA for continuous variables, with a *t*-test for post hoc pair-wise analysis, using Hochberg’s correction for multiple testing [32]. The chi-square test was used for categorical data. We also used supervised random forest classification models to identify the measures that contributed the most in the characterization of the clusters (discrimination of each cluster from all other clusters). In these random forest models, the cluster was a dichotomous outcome (cluster *k* vs. all other clusters), and all the variables included in the FAMD were the predictors. Please see supplementary methods for more detail about these random forest analyses (Additional file 2).

All statistical analyses were conducted with the R statistical software (R Foundation for Statistical Computing, Vienna, http://www-R-project.org) [29]. A *p*-value ≤0.05 was deemed statistically significant.

## Results

### Characteristics of the cohort

The key characteristics of the cohort are shown in Table 1. The average age was 68 ± 9 years and 28% of the patients were female. The average MMSE score was 25 ± 4. Parkinsonism and cognitive fluctuations were the most frequently reported clinical features (81% and 84%, respectively). Regarding the AD CSF biomarker profile, 11% of the patients had AD pathology (A+T+), 18% had an AD pathological change (A+T-), and 24% had amyloid-independent tau pathology (A-T+). Thus, 29% of patients can be categorized within the AD continuum according to the AT(N) framework. Atrophy was more frequent in the parietal lobe (57%) than in the medial temporal (33%) and frontal (39%) lobes.

**Table 1:** Characteristics of the whole cohort and DLB clusters

	Whole cohort	Cluster 1	Cluster 2	Cluster 3	Cluster 4	Between-cluster ANOVA
	(*N* = 107)	(*n* = 39)	(*n* = 25)	(*n* = 24)	(*n* = 19)	(*p*-value)
Age	68 (± 8.7)	70 (± 7.2)^b,d^	64 (± 7.7)^a,c^	71 (± 10)^a,d^	64 (± 6.9)^a,c^	0.001
Sex, *n* men (%)	77 (72.0%)	28 (71.8%)	21 (84.0%)^d^	21 (87.5%)^d^	7 (36.8%)^b,c^	0.001
Education, years mean (SD)	11 (± 3.8)	11 (± 2.8)^b,d^	8.2 (± 2.4)^a,c,d^	12 (± 3.5)^a,d^	15 (± 3.3)^a,b,c^	<0.001
Disease duration, years mean (SD)	4.3 (± 3.8)	4.2 (± 4.9)^d^	3.7 (± 2.7)^d^	3.5 (± 2.3)^d^	6.3 (± 3.8)^a,b,c^	0.013
MMSE score, mean (SD)	25 (± 4.0)	24 (± 3.9)^d^	22 (± 3.9)^d^	25 (± 3.8)^d^	28 (± 2.0)^a,b,c^	<0.001
**Core clinical features**						
Parkinsonism, *n* present (%)	87 (81 %)	33 (85 %)^c^	25 (100%)^c^	11 (46 %)^a,b,d^	18 (95 %)^c^	<0.001
Visual hallucinations, *n* present (%)	68 (64 %)	29 (74 %)^b^	8 (32 %)^a^	17 (71 %)	14 (74 %)	0.003
Cognitive fluctuations, *n* present (%)	90 (84 %)	39 (100 %)^b^	12 (48 %)^a,d^	20 (83 %)	19 (100 %)^b^	<0.001
Probable RBD, *n* present (%)	68 (64 %)	23 (59 %)	20 (80 %)	15 (62 %)	10 (53 %)	0.234
**CSF biomarkers**						
Aβ42, *n* abnormal (%)	31 (29 %)	16 (41 %)	6 (24 %)	8 (33 %)	1 (5 %)	0.037*
Total tau, *n* abnormal (%)	23 (21 %)	4 (10 %)^c^	0 (0 %)^c^	19 (79 %)^a,b,d^	0 (0 %)^c^	<0.001
p-tau, *n* abnormal (%)	38 (36 %)	11 (28 %)^c^	4 (16 %)^c^	23 (96 %)^a,b,d^	0 (0 %)^c^	<0.001
AD CSF profile, *n* abnormal (%)						<0.001
AD pathology	12 (11 %)	3 (8 %)	1 (4 %)	8 (33 %)	0 (0 %)	
AD pathological change	19 (18 %)	13 (33 %)	5 (20%)	0 (0 %)	1 (5%)	
Amyloid independent tau-pathology	26 (24 %)	8 (21 %)	3 (12 %)	15 (63 %)	0 (0 %)	
Normal	50 (47 %)	15 (38 %)	16 (64 %)	1 (4 %)	18 (95 %)	
**Visual rating scales**						
MTA, *n* abnormal (%)	35 (33 %)	23 (59 %)^b,c^	5 (20 %)^a^	3 (12 %)^a^	4 (21 %)	<0.001
GCA-F, *n* abnormal (%)	42 (39 %)	20 (51 %)^d^	11 (44 %)^d^	11 (46 %)^d^	0 (0 %)^a,b,c^	0.002
PA, *n* abnormal (%)	61 (57 %)	19 (49 %)	19 (76 %)^d^	19 (79 %)^d^	4 (21 %)^b,c^	<0.001
Fazekas, *n* high WMH burden (%)	29/92 (32%)§	15/32 (47%)^d^	6/24 (25%)	7/18 (39%)	1/17 (6%)^a^	0.018

No missing data was recorded for the rest of the variables. ^a^*p* < 0.05 compared to cluster 1. ^b^*p*<0.05 compared to cluster 2. ^c^*p*<0.05 compared to cluster 3. ^d^*p*<0.05 compared to cluster 4. ^§^Available data for Fazekas is *n* = 92. *Does not survive Hochberg’s correction in post hoc pair-wise comparisons. *Abbreviations: ANOVA* analysis of variance, *MMSE* Mini-Mental State Examination, *Aβ42* amyloid-beta 1-42, *p-tau* phosphorylated tau, *AD* Alzheimer’s disease, *MTA* medial temporal lobe atrophy, *GCA-F* global cortical atrophy-frontal subscale, *PA* posterior brain atrophy, *na* not applicable

### Factorial analysis of mixed data (FAMD)

**Fig. 2:**
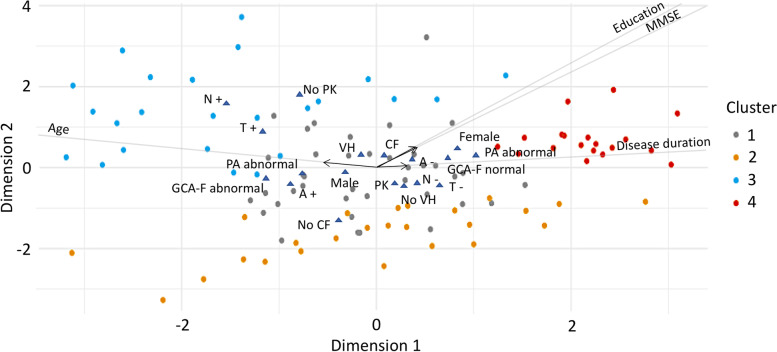
Dimension 1 vs. dimension 2. Continuous variables are depicted as arrows projecting lines (arrows represent the direction and degree of contributions). Categorical variables are depicted as triangles, which reflect variables’ centroids in the different levels of categorical variables. *Abbreviations: MMSE* Mini-Mental State Examination, *CF* cognitive fluctuations, *PK* parkinsonism, *VH* visual hallucinations, *A+* abnormal CSF Aβ42, *A-* normal CSF Aβ42, *T+* abnormal CSF p-tau, *T-* normal CSF p-tau, *N+* abnormal CSF total tau, *N-* normal CSF total tau, *GCA-F* global cortical atrophy-frontal brain atrophy subscale, *PA* posterior brain atrophy

**Fig. 3:**
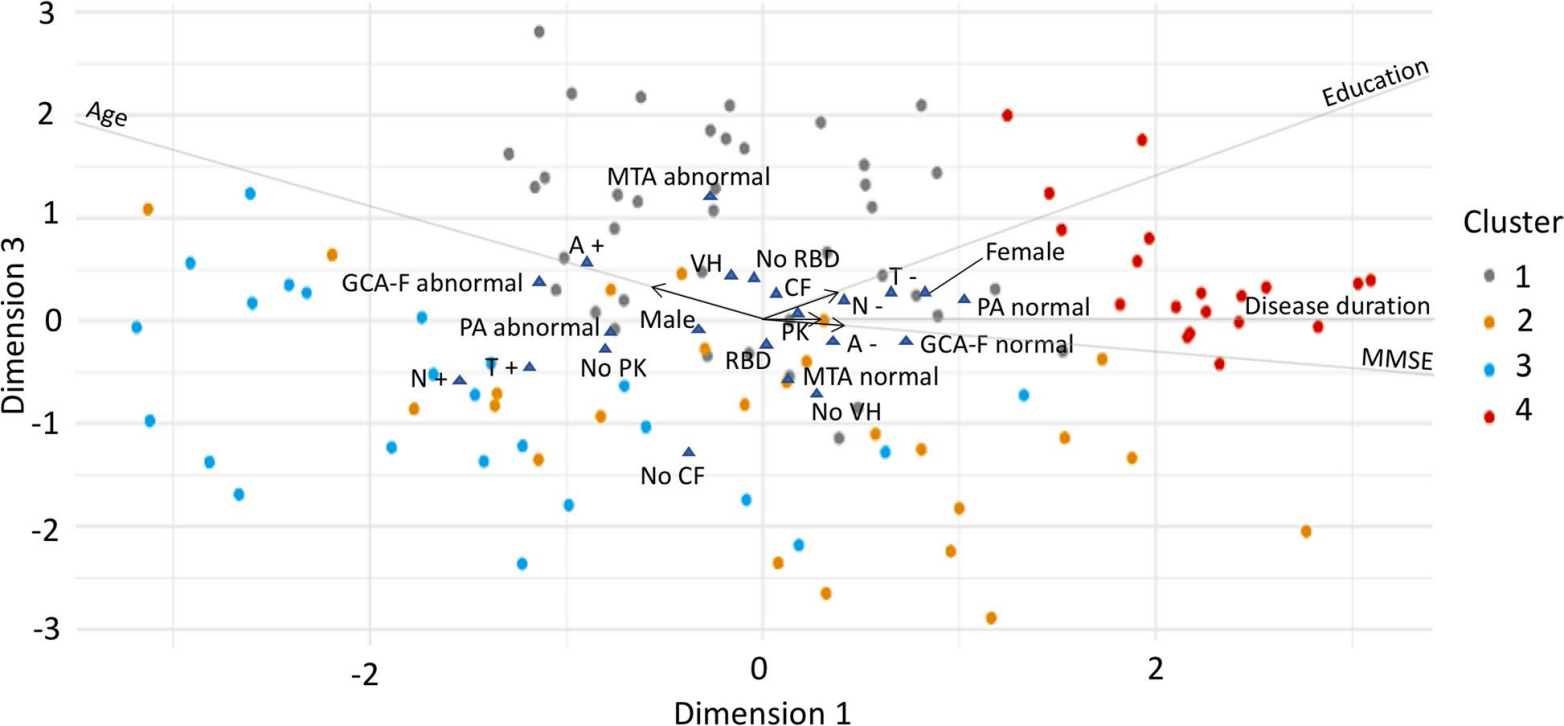
Dimension 1 vs. dimension 3. Continuous variables are depicted as arrows projecting lines (arrows represent the direction and degree of contributions). Categorical variables are depicted as triangles, which reflect variables’ centroids in the different levels of categorical variables. *Abbreviations: MMSE* Mini-Mental State Examination, *CF* cognitive fluctuations, *PK* parkinsonism*, VH* visual hallucinations, *RBD* REM sleep behavior disorder, *A+* abnormal CSF Aβ42, *A-* normal CSF Aβ42, *T+* abnormal CSF p-tau, *T-* normal CSF p-tau, *N+* abnormal CSF total tau, *N-* normal CSF total tau, *MTA* medial temporal lobe atrophy, *GCA-F* global cortical atrophy-frontal brain atrophy subscale, *PA* posterior brain atrophy

**Fig. 4:**
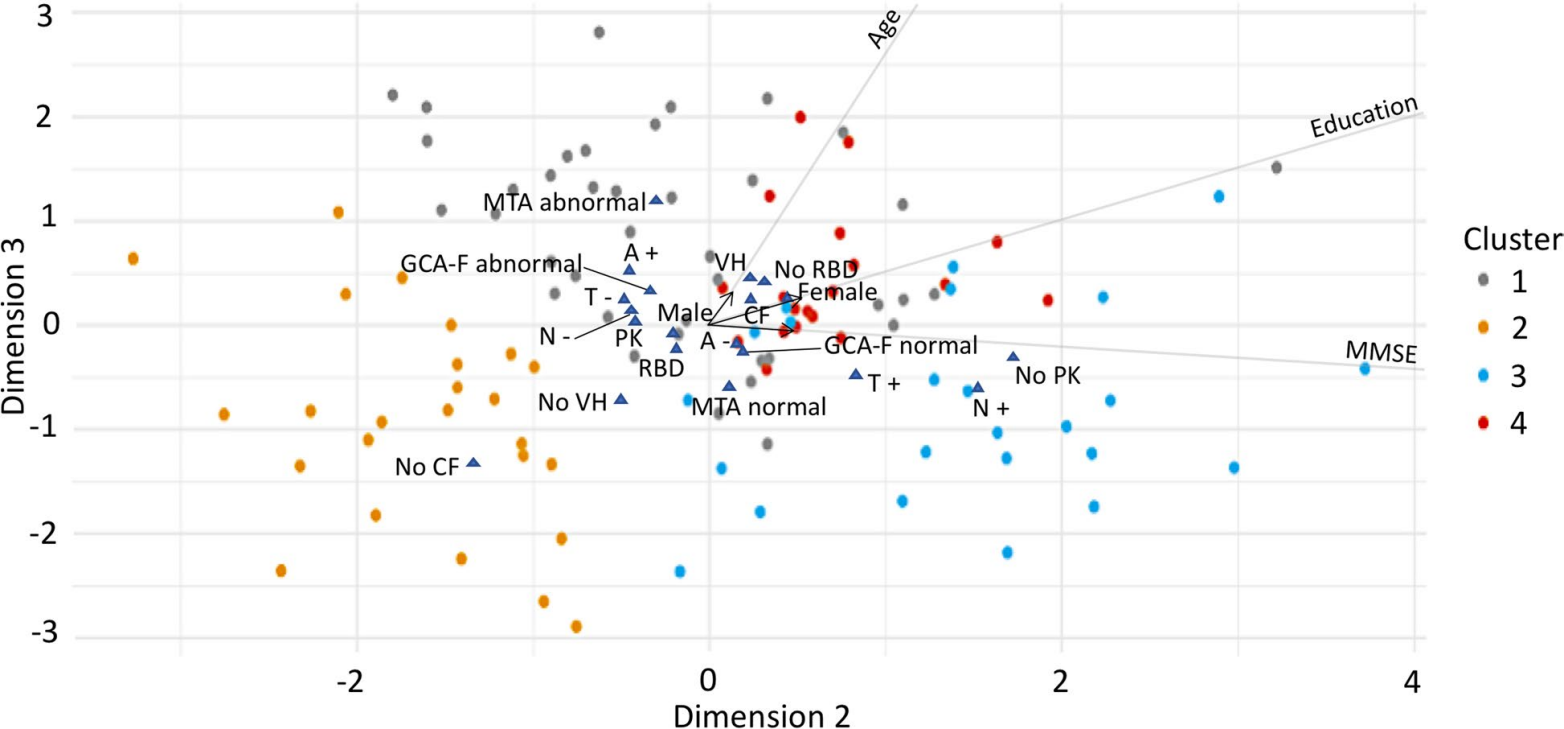
Dimension 2 vs. dimension 3. Continuous variables are depicted as arrows projecting lines (arrows represent the direction and degree of contributions). Categorical variables are depicted as triangles, which reflect variables’ centroids in the different levels of categorical variables. *Abbreviations: MMSE* Mini-Mental State Examination, *CF* cognitive fluctuations, *PK* parkinsonism, *VH* visual hallucinations, *RBD* REM sleep behavior disorder, *A+* abnormal CSF Aβ42, *A-* normal CSF Aβ42, *T+* abnormal CSF p-tau, *T-* normal CSF p-tau, *N+* abnormal CSF total tau, *N-* normal CSF total tau, *MTA* medial temporal lobe atrophy, *GCA-F* global cortical atrophy-frontal brain atrophy subscale

**Fig. 5:**
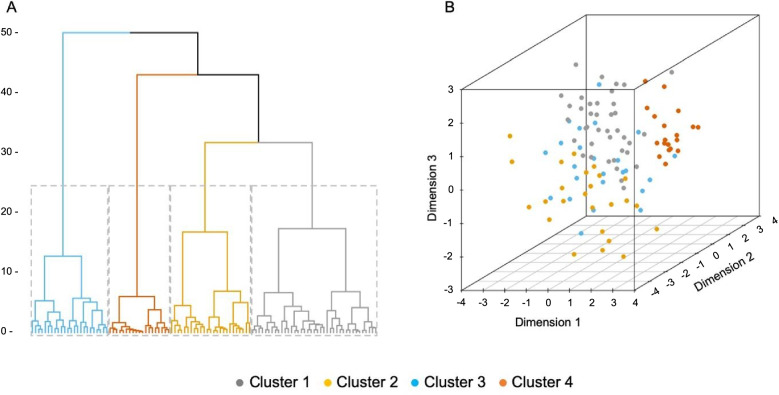
Dendrogram and clusters from the cluster analysis. **A** Dendrogram from the cluster analysis, with DLB patients depicted on the *x*-axis (each lower branch is a patient) and similarity depicted on the *y* axis (the shorter the distance along the axis, the greater the similarity). **B** Three-dimensional space generated by dimensions 1, 2, and 3 from the FAMD model. Dots represent the DLB patients colored by cluster [1 to 4] and distributed across the three-dimensional space

The FAMD model identified three dimensions that together explained 38% of the variance in the data. Table 2 shows variables’ contribution to these dimensions. Figures 2, 3, and 4 display the three dimensions pair-wise, and Fig. 5B displays all the three dimensions in a 3D space. The first dimension accounted for 15.7% of the variance and was mostly driven by atrophy in frontal and parietal lobes, CSF p-tau levels, and age. In particular, older patients had increased atrophy in frontal and parietal lobes and more often had abnormal CSF p-tau levels. In addition, CSF total tau levels, MMSE, years of education, CSF Aβ42 levels, sex, disease duration, and parkinsonism also contributed statistically significantly to the first dimension.

**Table 2:**
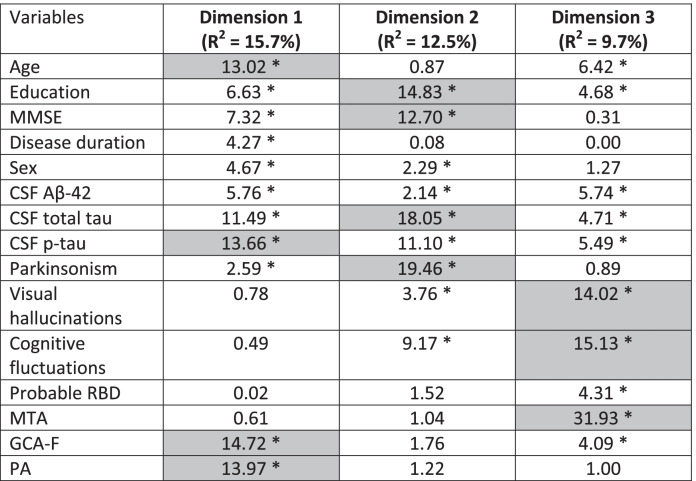
Contribution of each variable to the dimensions of the FAMD

Legend: Values represent the percentage of contribution of each variable to the total variation captured by each dimension. Gray-shadowed cells reflect the variables with highest contribution to each dimension. The asterisk (*) reflects the variables that contributed statistically significantly to each dimension. Abbreviations: *MMSE* mini-mental State examination; *CSF* cerebrospinal fluid; *Aβ* amyloid-beta; *p-tau* phosphorylated tau; *MTA* medial temporal lobe atrophy; *GCA-F* global cortical atrophy-frontal subscale; *PA* posterior brain atrophy; *FAMD* factorial analysis of mixed data

The second dimension accounted for 12.5% of the variance and was mostly driven by parkinsonism, CSF total tau levels, years of education, and MMSE. Patients with higher education showed higher MMSE scores despite more frequently having abnormal CSF total tau levels, and they had a lower frequency of parkinsonism. In addition, CSF p-tau levels, cognitive fluctuations, visual hallucinations, sex, and CSF Aβ42 levels also contributed statistically significantly to the second dimension.

The third dimension explained 9.7% of the variance and was mostly driven by atrophy in medial temporal lobes, cognitive fluctuations, and visual hallucinations. Patients with atrophy in the medial temporal lobes more often had cognitive fluctuations and visual hallucinations. In addition, age, CSF Aβ42, p-tau, and total tau levels, as well as years of education, probable RBD, and atrophy in frontal lobes, also contributed statistically significantly to the third dimension.

### Hierarchical clustering analysis

Subsequently, we clustered the patients using agglomerative hierarchical clustering analysis on the three dimensions from the FAMD model as the input data. Calinski-Harabasz (CH) values showed that four clusters (CH = 44.5) were more appropriate than two, three, or five clusters (CH < 42.0). Figure 5A shows the dendrogram from the cluster analysis, and Fig. 5B displays the distribution of the DLB patients colored by clusters 1 to 4.

Cluster 1 included 37% of the patients (*n* = 39), cluster 2 included 23% (*n* = 25), cluster 3 included 22% (*n* = 24), and cluster 4 included 18% (*n* = 19) of the DLB patients.

Table 1 shows key demographic and clinical data, as well as CSF and MRI measures for all clusters. Briefly, patients in cluster 1 were among the oldest and had intermediate levels of education, disease duration, and MMSE scores. Furthermore, all the patients in cluster 1 had cognitive fluctuations. Regarding AD CSF biomarkers, cluster 1 had the highest frequency of an AD pathological change (A+T-). As for regional brain atrophy, patients in cluster 1 had the highest frequency of medial temporal and frontal atrophy, showed intermediate levels of parietal atrophy, and had more often high WMH burden. The supervised random forest model showed that cognitive fluctuations, neurodegeneration markers (CSF total tau and regional atrophy), and age were the measures that best characterize this cluster (Supplementary Figure 1, Additional file 3).

Patients in cluster 2 had the lowest levels of education, MMSE scores, and frequency of visual hallucinations and cognitive fluctuations, and were among the clusters with younger age and shortest disease duration. Moreover, patients in cluster 2 had the highest prevalence of parkinsonism and, together with cluster 3, showed the highest frequency of parietal atrophy. The supervised random forest model showed that visual hallucinations and cognitive fluctuations, as well as education and age, were the measures that best characterize this cluster (Supplementary Figure 1, Additional file 3).

Patients in cluster 3 were the oldest, had intermediate levels of education and MMSE scores, had the shortest disease duration, and were the patients with the lowest frequency of parkinsonism. Furthermore, patients in cluster 3 had the highest levels of tau pathology, either in combination with a positive Aβ42 biomarker (AD pathology, A+T+) or independently of Aβ42 (amyloid-independent tau-pathology, A-T+). Additionally, cluster 3 patients had a significantly higher frequency of abnormal levels of total tau in CSF. The supervised random forest model showed that parkinsonism, and CSF total tau and p-tau were the measures that best characterize this cluster (Supplementary Figure 1, Additional file 3).

Patients in cluster 4 were among the youngest, had the lowest frequency of men, had the highest levels of education and MMSE scores, and had the longest disease duration. All patients in cluster 4 had cognitive fluctuations. All patients but one had a normal CSF AD biomarker profile (A-T-). Furthermore, patients in cluster 4 had the lowest frequency of parietal atrophy and WMH burden, and none of them had frontal atrophy. The supervised random forest model showed that education, frontal atrophy, and CSF p-tau were the measures that best characterize this cluster (Supplementary Figure 1, Additional file 3).

Clusters did not significantly differ in the frequency of probable RBD or abnormal levels of Aβ42 (irrespectively of p-tau levels). Yet, the difference in abnormal levels of amyloid-β emerged when considered together with the tau biomarker (AD pathology (A+T+) or AD pathological change (A+T-)), likely due to the contribution of tau-pathology.

## Discussion

In this study, we expanded the current knowledge about the biological heterogeneity within probable DLB by studying a relatively large biomarker cohort. We applied a method for multimodal subtyping on CSF biomarkers, structural MRI, and clinical and demographic measures, all of them combined. We identified four DLB subgroups that ranged from a cluster with almost no concomitant AD or cerebrovascular pathologies (cluster 4) to three clusters with various degrees of concomitant AD and/or cerebrovascular pathologies (clusters 1, 2, and 3), and as well different regional brain atrophy, clinical and demographic features.

Cluster 4 was characterized by the presence of virtually normal AD CSF biomarkers and a very low burden of cerebrovascular disease. Therefore, we suggest that the underlying pathology in this subgroup very likely is mainly α-synuclein-related. This subgroup included younger DLB patients with longer disease duration and better MMSE performance than the other 3 subgroups. Similarly, a previous study comparing DLB patients with and without concomitant AD pathology found that “pure” DLB subjects were younger and had higher MMSE scores [9]. Furthermore, our cluster 4 showed a slight predominance of women, while the whole cohort was mostly constituted by men. This sex distribution could be influenced by the sample site characteristics, since most of the patients in cluster 4 come from the Strasbourg center. Researchers from the Strasbourg center have recently a the predominance of women in DLB patients in France [33]. However, previous studies on sex differences in DLB have found mixed results. Some studies have demonstrated a predominance of women [33, 34], while other studies have shown an association between male sex and DLB [35, 36]. In relation to the core clinical features, all patients in cluster 4 had cognitive fluctuations, which is one of the most typical characteristics of DLB [37]. Additionally, cluster 4 showed the least regional brain atrophy, and all patients had normal total tau CSF levels. This implies no biomarker evidence of neurodegeneration in cluster 4, probably due to the absence of concomitant AD pathology and cerebrovascular disease [38–41]. Cluster 4 might thus reflect the purest DLB subtype in our cohort.

In contrast, the other three DLB subgroups showed varied degrees of concomitant AD or cerebrovascular pathologies. Our biological data based on CSF biomarkers and structural MRI suggest two different profiles. On the one hand, cluster 1 showed the highest frequency of AD pathological change (A+T-) and was characterized by medial temporal atrophy, and a high burden of cerebrovascular pathology. In addition, cluster 1 included older DLB patients. This combination of biological findings suggests a subtype with concomitant amyloid-β and cerebrovascular pathologies. The association between amyloid-β and older age [8], atrophy in the medial temporal lobe [7, 42–44], and cerebral amyloid angiopathy [45, 46] has been reported in previous studies. The novelty of our study is the identification of a subgroup that encapsulates all those features. Similarly, the limbic predominant subtype of AD also includes older patients with prominent medial temporal atrophy and a high burden of cerebrovascular pathology [11].

On the other hand, clusters 2 and 3 were characterized by a low frequency of medial temporal atrophy. What did characterize clusters 2 and 3 was the high frequency of posterior brain atrophy in both subgroups. The combination of posterior brain atrophy and sparing of medial temporal lobes describes the signature pattern of brain atrophy in probable DLB [10]. Cluster 3 included older DLB patients with the highest frequency of tau pathology, either in combination with amyloid-β pathology (hence reflecting AD pathology, A+T+) or in isolation (hence reflecting a non-AD pathological change, in this case, amyloid-independent tau-pathology, A-T+). In contrast, patients in cluster 2 were younger and most of them showed normal CSF AD biomarker levels (A–T–). Therefore, results from clusters 2 and 3 are again in agreement with recent studies showing that concomitant AD increases with age in probable DLB [8]. Furthermore, these results suggest that tau-related pathology can contribute to α-synuclein pathology either in isolation or in combination with amyloid-β, in DLB. One example of this is the high frequency of medial temporal atrophy in cluster 1, and the high frequency of posterior brain atrophy in clusters 2 and 3. Previous studies showed that medial temporal atrophy in DLB is associated with amyloid-β pathology [7, 42–44], while posterior brain atrophy is associated with the combined effect of amyloid-β and tau-related pathologies [42], matching the pattern of tau accumulation in the posterior cortex in positron emission tomography studies in DLB [26, 47, 48].

In addition, our data suggest a possible association between higher tau pathology and shorter disease duration. Cluster 3, the subgroup with the highest tau pathology, was among the clusters with the shortest disease duration. Previous studies showed that tau pathology is associated with a worse prognosis in DLB patients [49]. Cluster 2 was the other cluster among those with the shortest disease duration. Although tau levels were not high in cluster 2, patients in that subgroup had the lowest level of education (i.e., lower cognitive reserve). Hence, lower levels of tau pathology may be enough to lead to low MMSE scores in a shorter time, at younger ages, all of these being findings that characterize cluster 2. Altogether, disease duration was the shortest in both clusters 2 and 3, the two clusters with greater posterior brain atrophy, suggesting a more aggressive presentation of the disease. The subtype of AD with greater posterior brain atrophy has been proposed as the most aggressive presentation of the disease, possibly due to a higher frequency of concomitant AD and Lewy body pathology in that subtype of AD [11]. Similarly, Poulakis et al. reported two subgroups with posterior brain atrophy in AD, one with an older age (like cluster 3 in the current study) and one with a younger age (like cluster 2 in the current study) [50].

We observed statistically significant differences in the frequency of parkinsonism, visual hallucinations, and cognitive fluctuations across clusters that also had specific AD, cerebrovascular, and atrophy profiles. This result may have clinical implications. Clusters 1 and 4 showed the highest frequency of cognitive fluctuations, cluster 2 had the lowest frequency of visual hallucinations and cognitive fluctuations, and cluster 3 had the lowest frequency of parkinsonism. Hence, there seems to be an association between concomitant AD and cerebrovascular pathologies, and patterns of brain atrophy with clinical heterogeneity across subgroups of DLB patients. Although our current study did not primarily aim to investigate associations of specific pathological features with particular clinical core features, the FAMD and hierarchical cluster analysis showed that patients with a low frequency of parkinsonism usually have a higher frequency of abnormal CSF total tau and p-tau biomarkers. Our current data could help in guiding future studies that target specific clinic-pathological associations, perhaps using more detailed measures for both pathology and clinical features (as opposed to the dichotomized variables in the current study). Previous studies have found that amyloid-β, tau, and cerebrovascular pathologies are associated with a lower frequency of core clinical features [51, 52] and a less typical presentation of DLB [53, 54]. We thus highlight the relevance of these findings, since they suggest that probable DLB patients with concomitant AD or cerebrovascular pathologies may have a higher risk to be misdiagnosed. The different presentations of the disease with fast and slow progression rates also signify the clinical relevance of these DLB subgroups.

There were no differences in the frequency of probable RBD across clusters. Few studies have investigated the influence of AD or cerebrovascular pathologies upon probable RBD. Autopsy confirmed studies suggest that the burden of concomitant AD in DLB patients is inverse to the frequency of RBD, meaning that patients with a clinical history of RBD have less AD-related pathology and a higher frequency of diffuse Lewy body disease, and vice versa [55–57]. Recent biomarker studies found that higher levels of tau and cerebrovascular pathologies but not of amyloid-β were associated with a lower frequency of probable RBD [8, 41].

Interestingly, CSF amyloid-β was not one of the main drivers in the dimensions of the FAMD model, but it was still a significant contributor to all three dimensions. Opposite to having no contribution to the heterogeneity within DLB, this could indicate that CSF amyloid-β may be an underlying factor in all dimensions, contributing to more than one dimension at the same time. It is widely known that the contribution of tau pathology to brain atrophy and cognitive impairment is stronger than that of amyloid-β pathology [58–60], which could explain the not so strong contribution of CSF amyloid-β in our FAMD model.

## Limitations

Our study has some limitations. Firstly, we used a retrospective and cross-sectional cohort, and longitudinal studies will help to investigate the progression of these clusters over time. Secondly, our approach was data-driven, and thus, our current findings should be considered hypothesis generating—replication in independent cohorts is warranted. Thirdly, although our FAMD model included key factors that are known to explain DLB heterogeneity, other important biomarkers such as DaTSCAN, MIBG, and EEG and supportive clinical features like postural instability, syncope, systematic delusions, autonomic dysfunction, and others may explain the additional variance of DLB heterogeneity [1]. Those data were not available or were difficult to harmonize across centers in our current study. To move the field forward, future studies should investigate a wider range of variables in a prospective longitudinal cohort. Finally, given the multi-center nature of the current study, we cannot completely exclude that part of the heterogeneity investigated is due to differences between centers. However, it is difficult to separate between-center differences due to pure methodological reasons from between-center differences due to subpopulations with actual biological/phenotypical differences, as recently discussed [61]. Our finding showing that variables that are very well harmonized across centers (e.g., age, sex, education, MMSE) contributed to the dimensions in a similar manner than variables that are traditionally more difficult to harmonize (e.g., biomarkers) is reassuring and suggests that the influence of potential methodological differences across centers is likely not influencing our clusters. Indeed, combining data from several centers likely amplifies the heterogeneity in clinically diagnosed DLB patients, which was the main focus in our study. Therefore, cross-collaboration between specialized centers that used standard diagnostic procedures to reduce methodological differences across centers is a strength of our study, as well as the combination of both clinical and biomarker data, reflecting current clinical practice in DLB. We leveraged these rich multimodal data by using a method for multimodal subtyping for the first time in DLB, as far as we are aware.

## Conclusions

Our current study provides several insights on the contributors to the heterogeneity within probable DLB. The existence of subgroups of probable DLB has implications to clinical diagnosis. Furthermore, our preliminary data suggest the possibility that these different subtypes may have their own disease trajectories, and may need to be managed differently due to distinct combinations of core clinical features and concomitant AD and cerebrovascular pathologies. Therefore, future studies investigating longitudinal data of DLB subgroups are warranted. The recent development of the real-time quaking-induced conversion (RTQuIC), which accurately assesses α-synuclein pathology in vivo [62], may also help in elucidating longitudinal associations between α-synuclein, AD, and cerebrovascular pathologies in the near future. We believe that consideration of this heterogeneity is a first step into implementing personalized medicine approaches in DLB. Likewise, the presence of different subgroups of DLB may need to be accommodated in the design of future clinical trials in DLB. The recent approval of aducanumab by the US Food and Drug Administration posts the question about the potential benefit of anti-amyloid treatment in DLB patients with concomitant AD pathology. Our current findings suggest that the existence of different DLB subgroups with possibly different responses to anti-amyloid treatment should be considered.

## Supplementary Information

**Additional file 1 ***Supplementary table 1:* Overview of MRI parameters per center. *Supplementary table 2:* Overview of CSF procedures per center. These tables present detailed information about MRI parameters and CSF procedures per collaborating center.

**Additional file 2 ***Supplementary methods*. This file contains detail information of the supervised random forest classification model for the discrimination of each cluster from all other clusters.

**Additional file 3 ***Supplementary Fig. 1. Random forest classification models for the discrimination between a given cluster and all other clusters.* This figure depicts the lollipops of importance to report the results of the random forest models for the discrimination between a given cluster and all other clusters.

## Data Availability

The data that support the findings of this study are available from the E-DLB consortium but restrictions apply to the availability of these data, which were used under permission for the current study, and so are not publicly available. Anonymized data supporting the conclusions of the current study are available to qualified researchers on reasonable request to the E-DLB consortium.

## Abbreviations

AD: Alzheimer’s disease
ANOVA: Analysis of variance
Aβ: Beta-amyloid
CH: Calinski-Harabasz
CIBERNED: Centro de Investigación en Red-Enfermedades Neurodegenerativas
CSF: Cerebrospinal fluid
DLB: Dementia with Lewy bodies
E-DLB: European dementia with Lewy bodies consortium
ELISA: Enzyme-linked immunosorbent assay
FAMD: Factorial analysis of mixed data
FLAIR: Fluid-attenuated inversion recovery
GCA-F: Frontal brain atrophy
IIB-Sant Pau: Biomedical Research Institute-Sant Pau
MMSE: Mini-Mental State Examination
MRI: Magnetic resonance imaging
MTA: Medial temporal lobe atrophy
P-tau: Phosphorylated tau
PA: Posterior brain atrophy
RBD: REM sleep behavior disorder
REM: Rapid eye movement
RT-QuIC: Real-time quaking-induced conversion
SCI-lab: Sensory Cognitive Interaction Laboratory
WMH: White matter hyperintensity
