# Supplementary material for: Parsing heterogeneity within dementia with Lewy bodies using clustering of biological, clinical, and demographic data
Source: Alzheimers Res Ther. 2022 Jan 21;14:14. doi: 10.1186/s13195-021-00946-w (PMC8783432; doi:10.1186/s13195-021-00946-w)
Supplement: Supplementary file 1 — Additional file 1 Supplementary table 1: Overview of MRI parameters per center. Supplementary table 2: Overview of CSF procedures per center. These tables present detailed information about MRI parameters and CSF procedures per collaborating center. [file 13195_2021_946_MOESM1_ESM.docx]

**Supplementary table 1: Overview of MRI parameters per center**

| **Center** | **Number of DLB patients** | **MRI parameters** | |
| --- | --- | --- | --- |
| Amsterdam | 38 | Scanner, Field strength (T), TR(ms), TE(ms), TI(ms), FA, NoA, Resolution(mm) | GE Signa, 3T, 8, 3, 450, 12, (0.98x0.98x1) |
| Strasbourg | 38 | Scanner, Field strength(T), TR(ms), TE(ms), TI(ms), FA, NoA, Resolution(mm) | Siemens Verio, 3, 1900, 2.53, 900, 9, 1, (1, 1, 1) |
| Stockholm | 17 | Scanner, Field Strength (T), Resolution(mm) | Siemens Aera, 1.5T, (1, 1, 1) mm resolution  Siemens Avanto, 1.5T, (1.5, 1, 1) mm resolution  Siemens Avanto, 1.5T, (1.2, 1, 1) mm resolution  Siemens Avanto, 1.5T, (1.4, 1, 1) mm resolution  Siemens Symphony, 1.5T, (1.5, 1, 1) mm resolution  Siemens Symphony, 1.5T, (0.81, 1, 1) mm resolution  Siemens Trio, 3T, (1, 1, 1) mm resolution  Siemens Trio, 3T, (1.2, 1, 1) mm resolution  Siemens Trio, 3T, (0.9, 1, 1) mm resolution  Siemens Trio, 3T, (1.4, 1, 1) mm resolution |
| Brescia | 6 | Scanner, Field strength(T), TR(ms), TE(ms), TI(ms), FA, NoA, Resolution(mm) | Siemens Avanto, 1.5, 2050, 2.56, 1100, 15, 1, (0.5, 0.5, 1) |
| Barcelona | 5 | Scanner, Field strength (T), TR (ms), TE (ms), TI (ms), Resolution (mm) | Siemens Magnetom Aera, 3T, 2200ms, 2,23ms, 968ms, 1.1x1.1x1.2mm |
| Stavanger | 3 | Scanner, Field strength(T), TR(ms), TE(ms), FA, NoA, Resolution(mm) | Philips Intera, 1.5, 10, 4.6, 30, 2, (1.01, 1.01, 1)  Philips Intera, 1.5, 20, 16, 30, 1, (1.02, 1.02, 1)  GE Signa Excite, 1.5, 8.224, 3.144, 7, 1, (1, 1, 1) |

**Supplementary table 2: Overview of CSF procedures per center**

| **Center** | **Number of DLB patients** | **Centrifuging** | **Storage** | **Analysis Essay** | **Cut off values [ng/L]** |
| --- | --- | --- | --- | --- | --- |
| Amsterdam | 38 | Centrifuged at 1800 g for 10 min at 4°C | Aliquots of 0.5 mL stored in polypropylene tubes at -80°C | INNOTEST Double sandwich ELISAs | Aβ_42_: <550 t-tau: >375 p-tau: >52 |
| Strasbourg | 38 | Centrifuged at 1000g for 10 minutes at 4°C | Stored at -80°C | INNOTEST Double sandwich ELISAs | Aβ42: <550 T-tau: >400 P-tau: >80 |
| Stockholm | 17 | Centrifuged at 2000g for 10 minutes at 4°C | Aliquots of 0.5mL of 1mL stored in polypropylene tubes at -80°C | INNOTEST Double sandwich ELISAs | Aβ42: <550 T-tau: >400 P-tau: >80 |
| Brescia | 6 | Centrifuged at 3000g for 3 minutes at 4°C | Stored in polypropylene tubes at -80°C | INNOTEST Aβ42, Tau and P181-tau | Aβ42: <650  T-tau: >400  P-tau: >30 |
| Barcelona | 5 | Centrifuged at 2000g for 10 minutes at 4^o^C | Stored in polypropylene tubes at -80^o^C | INNOTEST Double sandwich ELISAs | Aβ42: <670  T-tau: >398  P-tau: >65 |
| Stavanger | 3 | Centrifuged at 2000g for 10 minutes at 4°C | Stored in polypropylene tubes at -80°C | Aβ42: Biosource Europe S.A.  t-tau: INNOTEST hTau  p-tau: INNOTEST Phos-pho-Tau (181) | Aβ42: <482 T-tau: >320 P-tau: >52 |
| Aβ42: Amyloid-β_42_. DLB: Dementia with Lewy bodies. ELISA: enzyme-linked immunosorbent assay. T-tau: total tau. P-tau: tau phosphorylated at threonine 181. | | | | | |
