# Supplementary material for: Parsing heterogeneity within dementia with Lewy bodies using clustering of biological, clinical, and demographic data
Source: Alzheimers Res Ther. 2022 Jan 21;14:14. doi: 10.1186/s13195-021-00946-w (PMC8783432; doi:10.1186/s13195-021-00946-w)
Supplement: Supplementary file 2 — Additional file 2 Supplementary methods. This file contains detail information of the supervised random forest classification model for the discrimination of each cluster from all other clusters. [file 13195_2021_946_MOESM2_ESM.docx]

**Supplementary methods**

We used supervised random forest classification models to identify the measures that contributed the most in the characterization of the clusters (discrimination of each cluster from all other clusters). In these random forest models, the cluster was a dichotomous outcome (cluster k *vs.* all other clusters), and all the variables included in the FAMD were the predictors. Hence, we performed four random forest classification models, one for each cluster versus all other clusters. Results from these analyses can be found in Supplementary Figure 1. Briefly, random forest is an ensemble method in machine learning based on growing of multiple decision trees via bootstrap aggregation (bagging). Each tree predicts a classification independently and votes for the corresponding class. The best model for each outcome variable is chosen from the majority of votes. The combination of bootstrap aggregation (Breiman, 1996) with random feature selection (Amit & Geman, 1997) in random forest is important to prevent data overfitting and increase the prediction power. Our random forest model included 5000 trees, providing an accurate estimation of the variables importance without introducing too much noise in the model due to the addition of redundant trees. Each of the trees was trained on randomly selected 70% of the data and subsequently tested on the unseen 30% of the data. A total of three variables were randomly selected and tested at each split, where the number of variables was defined by the square root of the total number of predictors in the model. The maximum depth of each tree was determined by the maximum number of nodes in each tree, ensuring at least one observation per node (i.e. trees were not truncated at a given depth). We conducted a random forest classification model (Liaw & Wiener, 2002), with cluster treated as the outcome variable, and age, years of education, MMSE scores, disease duration, sex (male vs. female), CSF Aβ42, p-tau and total tau levels, MTA, PA, and GCA-F scales (normal vs. abnormal), and parkinsonism, visual hallucinations, cognitive fluctuations, and probable RBD (absent vs. present) included as the predictors. We report the classification error as a measure of goodness of the model (out-of-the-bag estimated error rate, OOB-EER) (Breiman, 2001). When outcome variables are dichotomous, as it is our case, the error by chance is 50%. Therefore, a classification error below 50% is better than chance, with values closest to 0% denoting better classification performance, hence good reliability of the model. We also report the importance of the predictors as a measure of their contribution towards differentiating a given cluster from all other clusters. Higher important values denote stronger contribution to the prediction. This importance parameter was based on the mean decrease accuracy (MDA). Analyses were performed using R (www.R-project.org) version 3.2.4, with a p-value ≤0.05 deemed significant.

*References in supplementary methods:*

Amit, Y., Geman, D., 1997. Shape quantization and recognition with randomized trees. Neural Comput. 9, 1545e1588.

Breiman, L., 1996. Bagging predictors. Mach. Learn. 24, 123e140.

Breiman L. Random Forests. Machine Learning. 2001;45(1):5-32.

Liaw A, Wiener, M. Classification and regression by random forest. R News 2002;2(3):18–22.
