# Supplementary material for: Parsing heterogeneity within dementia with Lewy bodies using clustering of biological, clinical, and demographic data
Source: Alzheimers Res Ther. 2022 Jan 21;14:14. doi: 10.1186/s13195-021-00946-w (PMC8783432; doi:10.1186/s13195-021-00946-w)
Supplement: Supplementary file 3 — Additional file 3 Supplementary Fig. 1. Random forest classification models for the discrimination between a given cluster and all other clusters. This figure depicts the lollipops of importance to report the results of the random forest models for the discrimination between a given cluster and all other clusters. [file 13195_2021_946_MOESM3_ESM.docx]

**Supplementary Figure 1. Random forest classification models for the discrimination between a given cluster and all other clusters.**

The x-axis displays the importance of the variables in the differentiation between clusters, with higher values (dots to the right side) indicating a greater importance. MDA = mean decrease accuracy, MMSE = Mini-Mental State Examination, CSF = cerebrospinal fluid, CF = Cognitive fluctuations; PK = parkinsonism; VH = visual hallucinations; Aβ42 = amyloid-beta 1-42; MTA = medial temporal lobe atrophy; GCA-F = frontal brain atrophy; PA = posterior brain atrophy.
